# 

**Published:** 1901-08-03

**Authors:** 


**NOTICE TO CORRESPONDENTS.**

All MSS., Letters, Books for Review, and other matters intended for the Editor should be addressed The Editor, "The Hospital." The Hospital Building, 28 & 29 Southampton Street, Strand, W.C.

The Editor cannot undertake to return rejected MS., even when accompanied by stamped directed envelope.

**Notice to Advertisers and Subscribers.**

All Advertisements, Orders for Copies of The Hospital, and Business Communications should be addressed to **THE MANAGER (not to the Editor),** The Hospital, 28 & 29 Southampton Street, Strand, London, W.C.

The Cost of Subscription, post free, is as follows:—Per week, 2½d.; per quarter, 2s. 8d.; per half-year, 5s. 3d.; per annum, 10s. 6d. Foreign:—Per annum, 15s. 2d., payable, in all cases, in advance.

**NOTICE.—Vol. XXIX. of "The Hospital" (October 1900, to March, 1901), in handsome cloth binding is now on sale at the Office of this Journal, price, 7s. 6d. Cases for binding the Numbers contained in this Volume 1s. 6d. Index to Vol. XXIX., 2½d., post free.**

**The Monthly Part for August is now ready. Price 6d., by post 8d.**

### BOOKS RECEIVED.

Scott, Greenwood and Co.

"Practical X-Ray Work." By Frank T. Addyman, B.Sc.Lond., F.I.C.

Health Resorts Development Association.

"Weymouth: A Guide and Souvenir."

Baillière, Tindall and Cox.

"Introduction to Medical Jurisprudence." By William McCallin, M.D., B.Ch.

"An Atlas of the Anatomy of the Child." By D'Arcy Power, M.B.Oxon., F.R.C.S.

"The Law Relating to the Poor Law Medical Service and Vaccination." By M. Greenwood, M.D., LL.B.

"Public Health" Office.

"The Ethics of Preventive Medicine." By John C. McVail, M.D.

Rebman, Limited.

"The Influence of Hospital Isolation in Scarlet Fever." By C. Killick Millard, M.D., D.Sc.

John Bale, Sons, and Danielsson.

"Some Retrospects and Prospects in Surgery." By Reginald Harrison, F.R.C.S.

"When to Operate in Inflammation of the Appendix." By C. Mansell Moullin, M.D.Oxon., F.R.C.S.

John Falconer, Dublin.

"Some Results of the Nordrach Treatment in Ireland." By P. S. Hitchens, M.A., M.B., B.Ch. Oxon.

Watts and Co.

"Home Culture by Selection." By Henry Smith, M.D. (Jena).

Great Eastern Railway Company.

"Holidays in Eastern Counties." Edited by Percy Lindley.

"Rhineland." By Percy Lindley.

The Childhood Society.

"Lecture on Physiology for Teachers." By C. S. Sherrington, M.A., M.D. Camb., F.R.S.

W. B. Saunders and Co.

"Obstetric and Gynecologic Nursing."

Swan Sonnenschein and Co.

"A Treatise on Plague." By Major George S. Thomson, M.B., M.Ch., M.A., and John Thomson, M.R.C.S.I., L.R.C.P.I.

P. S. King.

"Local London, 1901-1902."

John Bale, Sons, and Danielsson.

"Sanatorium Treatment of Phthisis." By D. J. Chowry-Mutton, M.D., M.R.C.S., L.R.C.P.

### PARTICULARS OF AWARDS.

The following are the awards of the Committee of Distribution adopted by the Council of the Metropolitan Hospital Sunday Fund at the meeting on July 30th:—

### 31 GENERAL HOSPITALS.

Charing Cross Hospital, £1,050; French Hospital, £266 13s. 4d.; German Hospital, £441 13s. 4d.; Great Northern Central Hospital, £716 13s. 4d.; Guy's Hospital, £2,250; Hampstead Hospital, £200; Italian Hospital, £50; King's College Hospital, £1,250; London Hospital, £4,500; London Homœopathic Hospital, £458 6s. 8d.; Phillips' Memorial Homœopathic Hospital, £29 3s. 4d.; London Temperance Hospital, £666 13s. 4d.; Metropolitan Hospital, £616 13s. 4d.; Mildmay Hospital, £225; Miller Hospital and Royal Kent Dispensary, £250; North-West London Hospital, £341 13s. 4d.; Poplar Hospital, £433 6s. 8d.; Queen's Jubilee Hospital, £83 6s. 8d.; Royal Free Hospital, £875; St. George's Hospital, £1,937 10s.; SS. John and Elizabeth Hospital, £58 6s. 8d.; St. Mary's Hospital, £2,000; St. Thomas's Hospital, £966 13s. 4d.; Seamen's Hospital Society, £1,041 13s. 4d.; The Middlesex Hospital, £1,783 6s. 8d.; Tottenham Hospital, £258 6s. 8d.; University College Hospital, £1,133 6s. 8d.; Walthamstow, £70 6s. 8d.; West Ham Hospital, £375; West London Hospital, £600; Westminster Hospital, £1,291 13s. 4d.

### SPECIAL HOSPITALS.

#### 5 Chest Hospitals.

City of London Hospital for Diseases of the Chest, Victoria Park, £666 13s. 4d.; Hospital for Consumption, Brompton, £1,583 6s. 8d.; North London Consumption Hospital, Hampstead, £458 6s. 8d.; Royal Hospital for Diseases of the Chest, City Rd., £333 6s. 8d.; Royal National Hospital for Consumption (Ventnor), £291 13s. 4d.

#### 17 Children's Hospitals.

Alexandra Hospital for Hip Disease, W.C., £125; Banstead Surgical Home, £20 16s. 8d.; Barnet Home Hospital, £34 3s. 4d.; Belgrave Hospital for Children, S.W., £100; Cheyne Hospital for Incurable Children, S.W., £83 6s. 8d.; East London Hospital for Children, Shadwell, E.; £583 6s. 8d.; Evelina Hospital for Sick Children, Southwark, S.E., £291 13s. 4s.; Home for Incurable Children, Madia Vale, W., £66 13s. 4d.; Home for Sick Children, Sydenham, S.E., £108 6s. 8d.; Hospital for Sick Children, Great Ormond St., W.C., £833 6s. 8d.; North-Eastern Hospital for Children, Hackney Rd., N.E., £291 13s. 4d. Paddington Green Hospital for Children, W., £241 13s. 4d.; St. Mary's, Plaistow, E, £212 10s.; St. Monica's, Brondesbury, N.W., £87 10s.; Victoria Hospital for Children, King's Rd., Chelsea, S.W., £433 6s. 8d.; Victoria Home, Margate, £25; "The Vine," Sevenoaks, £33 6s. 8d.

6 Lying-in Hospitals.

British Lying-in Hospital, Endell St., W.C., £33 6s. 8d.; City of London Lying-in Hospital, City Rd., E.C., £45 16s. 8d.; Clapham Maternity Hospital, £33 6s. 8d.; East End Mother's Home, £75; General Lying-in Hospital, Lambeth, S.E., £35; Queen Charlotte's Lying-in Hospital, Marylebone Rd., W., £333 6s. 8d.

6 Hospitals for Women.

Chelsea Hospital for Women, S.W., £341 13s. 4d.; Hospital for Women, Soho Sq., W., £208 6s. 8d.; Grosvenor Hospital for Women and Children, Vincent Sq., S.W., £125; New Hospital, for Women, Euston Rd., W.C., £233 6s. 8d; Royal Hospital for Children and Women, Lambeth, S.E., £208 6s. 8d.; Samaritan Free Hospital, Lower Seymour St., W., £433 6s. 8d.

26 OTHER SPECIAL HOSPITALS.

Cancer Hospital, Brompton, S.W., £500: London Fever Hospital, Islington, N., £583 6s. 8d.; Gordon Hospital for Fistula, Vauxhall Bridge Rd., S.W., £22 10s.; St. Mark's Hospital for Fistula, City Rd., E.C., £125; National Hospital for the Diseases of the Heart, Soho Sq., W., £66 13s. 4d.; Female Lock Hospital, Harrow Rd., W., £208 6s. 8d.; Hospital for Epilepsy, Paralysis and other diseases of the Nervous System, Regent's Park, N.W., £41 13s. 4d.; National Hospital for the Paralysed and Epileptic, W.C., deferred; West End Hospital for Diseases of the Nervous System, £125; Central London Ophthalmic Hospital, Gray's Inn Rd., W.C., £100; Royal Eye Hospital, St. George's Circus, S.E., £175; Royal London Ophthalmic Hospital, City Rd., E.C., £625; Royal Westminster Ophthalmic Hospital, Charing Cross, W.C., £108 6s. 8d.; Western Ophthalmic Hospital, Marylebone Rd., W., £33 6s. 8d.; City Orthopædic Hospital, Hatton Garden, E.C., £166 13s. 4d.; National Orthopædic Hospital, Gt. Portland St., W., £104 3s. 4d.; Royal Orthopædic Hospital, Oxford St., W., £100; Royal Sea-Bathing Hospital, Margate, £291 13s. 4d.; St. John's Hospital for Diseases of the Skin, £66 13s. 4d.; St. Peter's Hospital for Stone, Covent Garden, £66 13s. 4d.; Central London Throat and Ear Hospital, Gray's Inn Rd., W.C., £31 13s. 4d.; London Throat Hospital, Great Portland St., W., £25; Metropolitan Ear, Nose and Throat Hospital, Grafton St., W., £12 10s.; Royal Ear Hospital, Frith St., W., £8 6s. 8d.; Dental Hospital of London, Leicester Sq., W.C., £104 3s. 4d.; National Dental Hospital, 149 Great Portland St., W., £20 16s. 8d.

29 CONVALESCENT HOSPITALS.

Metropolitan Convalescent Institution, Sackville St., W., £533 6s. 8d.; Bexhill Convalescent Home, Sackville St., W., £316 13s. 4d.; All Saints' Convalescent Hospital, Eastbourne, £333 6s. 8d.; Ascot Priory Convalescent Home, £58 6s. 8d.; Charing Cross Hospital Convalescent Home, Limpsfield, £58 6s. 8d.; Chelsea Hospital for Women Convalescent Home, St. Leonards, £37 10s.; Deptford Medical Mission Convalescent Home, Bexhill, £20 16s. 8d.; Mrs. Gladstone Convalescent Home, Mitcham, £25; Friendly Societies' Convalescent Home Dover, £16 13s. 4d.; Hahnemann Convalescent Home, Bournemouth, £25; Hanwell Convalescent Home, £16 13s. 4d.; Herbert Convalescent Home, Bournemouth, £16 13s. 4d.; Herne Bay Baldwin-Brown Convalescent Home, £25; Homœopathic Convalescent Home, Eastbourne, £16 13s. 4d.; King's College Hospital Convalescent Home, Hemel Hempstead, £54 3s. 4d.; Mrs. Kitto's Convalescent Home, Reigate, £58 6s. 8d.; London and Brighton Female Convalescent Home, nil; Mary Wardell Convalescent Home for Scarlet Fever, £75; Morley House Convalescent Home for Working Men, £175; Police Seaside Home, Brighton, £41 13s. 4d.; St. Andrew's Convalescent Hospital, Clewer, £116 13s. 4d.; St. Andrew's Convalescent Home, Folkestone, £208 6s. 8d.; St. John's Home for Convalescent and Crippled Children, Brighton, £75; St. Joseph's Convalescent Home, Bournemouth, £50; St. Leonards-on-Sea Convalescent Home for Poor Children, £83 6s. 8d.; St. Mary's Convalescent Home, Shortlands, £15; St. Michael's Convalescent Home, Westgate-on-Sea, £29 3s. 4d.; Seaside Convalescent Hospital, Seaford, £83 6s. 8d.; Warley Convalescent Cottage Hospital, Essex, £[illegible] 6s. 8[illegible].

18 Cottage Hospitals.

Beckenham Cottage Hospital, £45 16s. 8d.; Blackheath and Charlton Cottage Hospital, £62 10s.; Bromley, Kent, Cottage Hospital, £83 6s. 8d.; Bushey Heath Cottage Hospital, £37 10s.; Canning Town Cottage Hospital, £41 13s. 4d.; Chislehurst, Sidcup, and Cray Valley Cottage Hospital, £41 13s. 4d.; Eltham Cottage Hospital, £33 6s. 8d.; Enfield Cottage Hospital, £45 16s. 8d.; Epsom and Ewell Cottage Hospital, £54 3s. 4d.; Hounslow Cottage Hospital, £22 10s.; Livingstone Cottage Hospital, £65; Mildmay Cottage Hospital, £41 13s. 4d.; Reigate and Redhill Cottage Hospital, £66 13s. 4d.; Sidcup Cottage Hospital, £31 13s. 4d.; Tilbury (Passmore Edwards) Cottage Hospital, £33 6s. 8d.; Willesden (Passmore Edwards) Cottage Hospital, £37 10s.; Wimbledon Cottage Hospital, £33 6s. 8d.; Woolwich and Plumstead Cottage Hospital, £29 3s. 4d.

7 INSTITUTIONS.

Establishment for Gentlewomen, Harley St., £91 13s. 4d.; St. Saviour's Hospital and Nursing Home, £100; National Sanatorium for Consumption, Bournemouth, £75; Invalid Asylum, Stoke Newington, £41 13s. 4d.; Firs Home, Bournemouth, £16 13s. 4d.; St. Catherine's Home, Ventnor, £25; Friedenheim Home for the Dying, £208 6s. 8d.

55 DISPENSARIES.

Battersea Provident Dispensary, £70 16s. 8d.; Bloomsbury Provident Dispensary, £10; Brixton, &c. Dispensary, £40 Brompton Provident Dispensary, £18 6s. 8d.; Camberwell Provident Dispensary, £58 6s. 8d.; Camden Provident Dispensary, £10 16s. 8d.; Chelsea, Brompton and Belgrave Dispensary, £33 6s. 8d.; Chelsea Provident Dispensary, £10; City Dispensary, £91 13s. 4d.; City of London and East London Dispensary, £18 6s. 8d.; Clapham General and Provident Dispensary, £20; Deptford Medical Mission, £15; Eastern Dispensary, £23 6s. 8d.; East Dulwich Provident Dispensary, £26 13s. 4d.; Farringdon General Dispensary, £36 13s. 4d.; Finsbury Dispensary, £33 6s. 8d.; Forest Hill Dispensary, £31 13s. 4d.; Greenwich Provident Dispensary, £19 3s. 4d.; Hackney Provident Dispensary, £9 3s. 4d.; Hampstead Provident Dispensary, £33 6s. 8d.; Holloway and North Islington Dispensary, £43 6s. 8d.; Islington Dispensary, £41 13s. 4d.; Kensal Town Provident Dispensary, £8 6s. 8d.; Kensington Dispensary, £47 10s.; Kilburn, Maida Vale, and St. John's Wood Dispensary, £31 13s. 4d.; Kilburn Provident Medical Institution, £25; Leman Street Provident Dispensary, nil; London Dispensary, £12 10s.; London Medical Mission, Endell St., £83 6s. 8d.; Margaret St. Infirmary for Consumption, nil; Metropolitan Dispensary, £38 6s. 8d.; Notting Hill Dispensary and Maternity (Provident), £7 10s.; Paddington Provident Dispensary, £25; Portland Town Dispensary, £14 3s. 4d.; Public Dispensary, £39 3s. 4d.; Queen Adelaide's Dispensary, £16 13s. 4d.; Royal General Dispensary, £20 16s. 8d.; Royal Pimlico Provident Dispensary, £50; Royal South London Dispensary, £33 6s. 8d.; St. George's and St. James's Dispensary, £40; St. George's, Hanover Sq., Provident Dispensary, £25; St. John's Wood and Portland Town Provident Dispensary, £26 13s. 4d.; St. Marylebone General Dispensary, £25; St. Pancras and Northern Dispensary, £25; South Lambeth, Stockwell and North Brixton Dispensary, £33 6s. 8d.; Stamford Hill, Stoke Newington, &c., Dispensary, £48 6s. 8d.; Tower Hamlets Dispensary, £33 6s. 8d.; Walworth Provident Dispensary, £10; Wandsworth Common Provident Dispensary, £6 13s. 4d.; Westbourne Provident Dispensary and Maternity, £10 16s. 8d.; Western Dispensary, £46 13s. 4d.; Western General Dispensary, £104 3s. 4d.; Westminster General Dispensary, £29 3s. 4d.; Whitechapel Provident Dispensary, £19 3s. 4d.; Woolwich Provident Dispensary, £10.

### HOSPITAL MEETINGS.

### EAST LONDON HOSPITAL FOR CHILDREN.

### Scraps and Gleanings.

The estimated cost of the new laundry and other extensions at the Warneford Hospital, Leamington, is £2,000.

Messrs. Brand and Co., Limited, have been appointed purveyors of Concentrated Beeftea to H.M. King Edward VII.

The fifteenth annual parade of friendly societies at Dursley was held early in July on behalf of the funds of the Gloucester Infirmary. By means of these parades the sum of something like £160 has been collected for the Infirmary.

At a meeting held recently in Gloucester, with the object of taking steps to erect a sanatorium for the counties of Gloucestershire, Somersetshire, and Wiltshire for the open-air treatment of consumption, a resolution was unanimously agreed to cordially support the scheme and to do all possible to help it forward by assisting to raise the necessary funds.

### The Student of Medicine.

### Appointments.

**G. W. Brabyn**, L.R.C.S., L.R.C.P.Edin., L.F.P.S.Glasg., appointed one of the Public Vaccinators for the South Wimbledon District of the Kingston Union. **Harold Collinson**, M.B.Lond., M.R.C.S., L.R.C.P., appointed Resident Casualty Officer to the General Infirmary, Leeds. **E. E. Crawshaw**, M.R.C.S., L.R.C.P., appointed Resident Obstetric Officer to the General Infirmary, Leeds. **A. Davidson**, M.D., appointed Port Medical Officer at Singapore. **M. P. Dunlea**, M.D., M.Ch.R.U.I., appointed Certifying Factory Surgeon for the Riverstown District, co. Cork. **Marian Erskine**, L.R.C.P. and S., appointed Junior Assistant Medical Officer to the Edinburgh Hospital and Dispensary for Women and Children. **Hugh Faulkner**, M.B., Ch.B.Edin., appointed House Physician to the General Hospital, Birmingham. **Hunter Finlay**, M.D., L.F.P.S.G., L.M., J.P., appointed Medical Officer to the Hospital at Angledool, New South Wales. **Ernest E. Glynn**, M.R.C.S., L.R.C.P.Lond., appointed Honorary Assistant Physician to the Liverpool Hospital for Consumption. **W. Watson Griffin**, F.R.C.S., appointed Ophthalmic Surgeon to the Sussex County Hospital, Brighton. **A. S. F. Grunbaum**, M.A.Cantab., M.D., M.R.C.P., appointed Honorary Assistant Physician to the Liverpool Hospital for Consumption. **W. B. Hayes**, M.D.R.U.I., M.Ch., appointed Certifying Factory Surgeon for the Union of Tralee, co. Kerry. **Sir Francis Laking**, K.C.V.O., M.R.C.P.Lond., appointed Consulting Physician to the Victoria Hospital for Sick Children, Chelsea. **E. J. Maclean**, M.D.Edin., M.R.C.P.Lond., appointed Gynæcologist to the Cardiff Infirmary. **J. C. Maddever**, M.D.Glasg., C.M., appointed Certifying Factory Surgeon for the Brownhills and Aldridge District of Staffordshire. **G. Norman Meachen**, M.B., B.S.Lond., M.R.C.P.Edin., M.R.C.S., appointed Clinical Assistant to the Hospital for Diseases of the Skin, Blackfriars. **Martin R. Morrissey**, L.R.C.P., L.R.C.S.Edin., appointed Medical Officer for the Blessington No. 2 Dispensary District. **J. T. C. Nash**, M.D.Edin., D.P.H.Cantab., appointed Medical Officer of Health for the Borough of Southend-on-Sea. **D. J. O'Brien**, L.R.C.P., L.R.C.S.Irel., appointed Certifying Factory Surgeon for the Durrow Dispensary District, Queen's County. **T. Pickering Pick**, F.R.C.S.Eng., appointed Consulting Surgeon to the Victoria Hospital for Sick Children, Chelsea. **W. G. Pritchard**, L.R.C.P., L.R.C.S.Edin., L.F.P.S.Glasg., appointed Medical Officer and Public Vaccinator to the No. 2 District of the Bangor and Beaumaris Union; also Factory Surgeon for the Bethesda Urban District and the civil parishes of Llanllechid and Llandegai. **Geo. Rorie**, M.B., M.Ch.Edin., appointed Senior Assistant Medical Officer to the Dorset County Asylum. **Edgar Stevenson**, M.D., appointed Demonstrator in Ophthalmology at University College, Liverpool. **H. Hyslop Thomson**, M.D., M.C., appointed Resident Medical Officer to the Consumption Sanatoria, Bridge-of-Weir, Renfrewshire. **A. S. Tredinnick**, L.R.C.P.Lond., M.R.C.S.Eng., appointed Certifying Factory Surgeon for the Melbourne District of Derbyshire. **J. W. Watkins**, M.B., B.Ch.Dub., appointed Certifying Factory Surgeon for the Bolsover District of the County of Derby. **Mrs. D. Chalmers Watson**, M.D., appointed Senior Assistant Medical Officer to the Edinburgh Hospital and Dispensary for Women and Children. **J. Howard Wilkinson**, L.R.C.P.Lond., M.R.C.S.Eng., D.P.H.Oxon., appointed an Honorary Surgeon to the Guest Hospital, Dudley.

